# INCAWrapper: a Python wrapper for INCA for seamless data import, -export, and -processing

**DOI:** 10.1093/bioadv/vbae100

**Published:** 2024-07-04

**Authors:** Matthias Mattanovich, Viktor Hesselberg-Thomsen, Annette Lien, Dovydas Vaitkus, Victoria Sara Saad, Douglas McCloskey

**Affiliations:** Novo Nordisk Foundation Center for Biosustainability, Technical University of Denmark, Lyngby, 2800, Denmark; Novo Nordisk Foundation Center for Basic Metabolic Research, University of Copenhagen, Copenhagen, 2200, Denmark; Novo Nordisk Foundation Center for Biosustainability, Technical University of Denmark, Lyngby, 2800, Denmark; Novo Nordisk Foundation Center for Biosustainability, Technical University of Denmark, Lyngby, 2800, Denmark; Novo Nordisk Foundation Center for Biosustainability, Technical University of Denmark, Lyngby, 2800, Denmark; Novo Nordisk Foundation Center for Biosustainability, Technical University of Denmark, Lyngby, 2800, Denmark; Novo Nordisk Foundation Center for Biosustainability, Technical University of Denmark, Lyngby, 2800, Denmark; BioMed X Institute, Artificial Intelligence, Heidelberg, Baden-Württemberg, 69120, Germany

## Abstract

**Motivation:**

INCA is a powerful tool for metabolic flux analysis, however, import and export of data and results can be tedious and limit the use of INCA in automated workflows.

**Results:**

The INCAWrapper enables the use of INCA purely through Python, which allows the use of INCA in common data science workflows.

**Availability and implementation:**

The INCAWrapper is implemented in Python and can be found at https://github.com/biosustain/incawrapper. It is freely available under an MIT License. To run INCA, the user needs their own MATLAB and INCA licenses. INCA is freely available for noncommercial use at mfa.vueinnovations.com.

## 1 Introduction

The study of metabolic flux is crucial for metabolism research. Metabolic flux data has led to important findings in diverse fields, including cancer research (Antoniewicz 2018), cell factory design (Gurdo *et al.* 2023), and basic research of autotrophic systems (Jazmin *et al.* 2014). Currently, carbon-13 Metabolic Flux Analysis (13C-MFA) is the standard approach to determine a large number of intracellular fluxes from experimental data (Antoniewicz 2018), but it involves laborious experimental- and computational work. Several software can estimate the intracellular fluxes from stable isotope labeling experiments; including INCA (Rahim *et al.* 2022), 13CFlux2 (Weitzel *et al.* 2013), and OpenFlux (Quek *et al.* 2009). While these examples are either closed source- or MATLAB-based applications, open source packages exist, usually written in Python such as FreeFlux (Wu *et al.* 2023), but have not been thoroughly tested and applied by the community. Here, we present a Python wrapper for INCA that includes additional tools to facilitate its use and expand its application. INCA was selected as the basis for this tool since it can calculate fluxes based on labeling experiments with other elements than carbon, e.g. nitrogen-15, deuterium, or combinations.

INCA can be operated via a graphical user interface (GUI) or through MATLAB code. The interface is well documented and the authors recommend using it to get familiar with INCA’s functionalities. INCA offers a spreadsheet import option for the isotopologue distribution data. However, this functionality is limited to importing one metabolite at a time for each labeling experiment. Thus, entering all the required information and data into the interface is a rather time-consuming, manual process, which makes it difficult to analyze a larger number of datasets.

Python is the most commonly used programming language in science, making it highly beneficial for scientific software to be interoperable with Python. Interoperability allows researchers to integrate existing tools into (automated) workflows. These considerations led to the creation of the INCAWrapper Python package.

The INCAWrapper enables importing data, running INCA, exporting the results, and doing model diagnostics without leaving Python. Thus, the INCAWrapper is an effective tool to integrate labeling-based MFA using INCA into Python-based workflows.

## 2 Implementation

**Figure 1.**
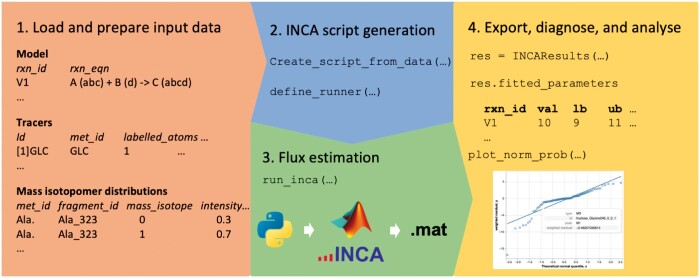
Workflow. The steps from data input to output visualization. (1) Defined input tables provide the information required for an MFA. (2) The INCAWrapper writes a script for the analysis. (3) Communication with INCA and MATLAB to run the MFA. (4) The output is reimported into Python and can be used for further analysis. This entire workflow can be performed without leaving Python.

The INCAWrapper wraps around INCA on both sides, providing functionality to input data and read the output into interoperable and open file formats (CSV files). Upstream, it allows users to specify their model and data in CSV or Excel files, which are then loaded into Python to set up the metabolic model. The INCAWrapper can then execute one or more of INCA’s algorithms “Estimate,” “Continuate,” and “Montecarlo.” Downstream, this package can read an INCA output file into a Python object which contains all the produced results. The Python object class provides methods to save the results and conduct simple analysis, model diagnostics, and visualizations, and to return the results as Pandas DataFrames, which can be used in custom visualizations, -analysis, or -pipelines (see Figure 1).

### 2.1 Data requirements

The INCAWrapper requires the following information to conduct labeling-based MFA:

1. a metabolic model (with mapped atom transitions), e.g. A (abc) + B (d) -> C (abcd)
2. Mass isotopologue distributions
3. Information about the tracers

To obtain absolute flux values, users must additionally supply one or more measured uptake and/or secretion rates (Antoniewicz 2018).

Data is input as Pandas DataFrames, which can be generated from CSV or Excel files loaded into Python. The structure of these data frames follows the Tidy data format. To avoid mistakes, the input data structure is validated using Pandera, ensuring that the data types and formats are compatible with INCA.

### 2.2 The core of the INCAWrapper

The INCAWrapper runs INCA through a MATLAB script. This script defines the model, the data, the settings, and what algorithms to execute. Thus, it is a metaprogramming tool, which transforms information from Pandas DataFrames into script blocks. The INCA script can be exported, which can be shared to reproduce the results.

The INCAWrapper provides a high-level API, where a fully executable INCA script is generated in two lines of code from loaded and correctly formatted input data. This script can be modified to change the default options or further specify the model (e.g. define symmetric metabolites). Additionally, a low-level API for more fine-grained control is provided.

INCA outputs a single MATLAB struct object consisting of several tables. The INCAWrapper can parse this MATLAB object into a Python object, which can export the individual data tables and has methods for simple diagnostics and plots.

The INCAWrapper can also be used to process data to be imported into the INCA GUI, and subsequently export the output data into Python for further processing.

To demonstrate the capabilities of the INCAWrapper, we prepared tutorials that can be found in the GitHub repository and on the documentation page (https://incawrapper.readthedocs.io/en/latest/examples/index.html). We sought to make these tutorials as realistic as possible. Therefore, we used published data from 13C labeling experiments, showed how to wrangle the data into the correct format for the INCAWrapper, conduct flux estimation, and evaluate results. They are provided as Jupyter Notebooks to be directly reusable.

## 3 Testing and validation

In order to ensure that the provided software is reliable, the INCAWrapper codebase is thoroughly tested and the results were validated in two separate ways: unit tests, and validation against an analysis performed using the GUI.

The individual Python functions are routinely tested through unit tests (https://github.com/biosustain/incawrapper/tree/main/tests), which ensures that each function produces the expected output. These unit tests improve maintainability, and thereby increase the lifespan of the software.

As a Python wrapper for INCA, the INCAWrapper and the GUI execute the same functions. To test this, we created a validation dataset using INCA’s simulation functionality. The validation dataset utilizes a small toy model with five reactions. We analyzed the datasets entirely through the INCA GUI and using the INCAWrapper. We used the MCMC sampling algorithm in INCA, and subsequently compared the samples from the GUI and the INCAWrapper, using a Hotteling’s *t*^2^ test. This test revealed no evidence that the mean flux distributions of the two sets of samples were different (*P* = .364). (https://incawrapper.readthedocs.io/en/latest/examples/validation_toy_model.html).

## 4 Discussion

A drawback of the INCAWrapper is that the input data does not utilize any standardized file format. This is because, to the best of our knowledge, there is no widely accepted standard for labeling-based MFA. Considered were SBML and the Flux Markup Language (FluxML) (Beyß *et al.* 2019). However, it has earlier been noted in Beyß *et al.* that SBML is not sufficient for labeling-based MFA data, and we found FluxML’s installation and use requirements to be too advanced for nonexpert users. We therefore decided to use tabular data files.

INCAWrapper improves the FAIRness of labeling-based MFA by using tabular data for both in- and output, which enables the integration of labeling-based MFA into larger data processing pipelines. FAIRness could be further enhanced by using easy-to-use community standards for in- and output. Furthermore, by remaining in one Python environment, the calculated fluxes can easily be used to constrain COBRApy models. Sampling these constrained models can extrapolate the MFA flux prediction to genome scale.

All functionalities cannot be used without licenses to INCA and MATLAB. However, the output of INCA can be loaded into Python using the INCAWrapper without requiring any licenses. Thus, the INCAWrapper introduces an open-source interface that improves the reusability of the results of flux estimation done by INCA.

For optimal reproducibility, we recommend that users publish, among all the generally required information (Crown and Antoniewicz 2013), both the in- and output CSV files and the MATLAB files generated by INCA, which are very well structured and include all the necessary information to interpret the fit.

INCA provides a plethora of functionalities and the INCAWrapper currently does not cover all of them. For example, it does not cover input of NMR data, or using the experimental design algorithms. These functionalities require interaction with the INCA GUI.

In conclusion, the INCAWrapper simplifies the labeling-based MFA workflow and enables higher throughput and a more convenient interface for researchers using Python.

## Data Availability

No new data were generated or analysed in support of this research.
